# The Role of Cytokines in Inflammatory Bowel Disease

**DOI:** 10.1155/S0962935194000013

**Published:** 1994

**Authors:** G. Radford-Smith, D. P. Jewell

**Affiliations:** Gastroenterology Unit Radcliffe Infirmary Oxford UK

## Abstract

Cytokines play an important role in the development and persistence of the inflammatory lesions seen in Crohn's disease and ulcerative colitis. This review discusses the current thinking of the role of cytokines in chronic intestinal inflammation including the involvement of immunoregulatory cytokines within the Th1 and Th2 subsets.


Invited Review

Mediators of Inflammation 3, 3–9 (1994)

### The role of cytokines in inflammatory bowel disease

G. Radford-Smith and D. P. Jewell*

Gastroenterology Unit, Radcliffe Infirmary, Oxford, UK

CA Corresponding Author

**CYTOKINES play an important role in the development and persistence of the inflammatory lesions seen in Crohn's disease and ulcerative colitis. This review discusses the current thinking of the role of cytokines in chronic intestinal inflammation including the involvement of immunoregulatory cytokines within the Th1 and Th2 subsets.**

**Key words:** Ulcerative colitis (UC), Crohn's disease (CD), Cytokines

### Introduction

Cytokines are powerful peptide molecules secreted by a number of different cell lineages, including cells of the immune system. Although originally thought to be highly specific in their actions, numerous studies over the past 5 years have demonstrated a large amount of pleiotropism and redundancy within the cytokine network.[1] Therefore, caution should be applied when evaluating the role of these molecules in various inflammatory disorders, whether of established or unknown aetiology.

Crohn's disease (CD) and ulcerative colitis (UC) are both good examples of chronic inflammatory disease of unknown aetiology.[2] CD can affect any part of the intestine from mouth to anus, with the inflammation often being transmural and the infiltrate characterized by macrophages and lymphocytes predominantly. The inflammatory process in UC is confined to the mucosa and superficial submucosa of the large bowel. The inflammatory infiltrate comprises neutrophils and lymphocytes, as well as other constituents of both acute and chronic inflammation.

The study of these two diseases has been hampered by the lack of a good animal model, although the recent progress with transgenic[3] and knockout animals[4–6] may prove useful. The investigation of cytokines has been carried out at great pace, but possibly at the expense of clarity and uniformity of method. The aim of this review is to clarify our current understanding of cytokines in chronic intestinal inflammation and establish whether current work is heading in the right direction.

### Animal Models

The use of animal models for inflammatory bowel disease (IBD) initially relied on some form of chemically induced injury to the intestine. This sets up an acute response, without always producing the more typical chronic relapsing and remitting form of inflammation seen in UC and CD.[7] However, some of these studies have been useful in studying the role of cytokines. Gurbindo *et al.*[8] have used the TNBS-induced colitis model in rats to assess interleukin 2 (IL2) activity of lamina propria mononuclear cells (LPMC) in the inflamed colon. The LPMC isolated from animals with acute colitis showed significantly less IL2 activity when compared with animals with chronic or resolved disease. In addition, LPMC from animals with acute disease responded poorly to mitogen stimulation (concanavalin A, 4 μg/ml) when compared with the other two groups. This poor response may be secondary to preactivation *in vivo*, leaving the cells refractory to further stimulation. There may also be increased uptake of IL2 by a greater number of immune cells bearing IL2 receptors on the cell surface. Expression of these receptors and other activation antigens goes up in the acute phase of the disease in humans.[9,10] Finally, the differences observed may be a reflection of different lymphocyte subsets occupying the gut mucosa at the various stages of the colitis model, and in turn, these subsets may produce differing cytokine profiles. This point has not been addressed as yet.

Using embryonic stem cell technology, a number of groups have developed mouse mutants specifically deficient in a given cytokine, thus allowing a closer look at the role of that molecule *in vivo*. Disruption of the IL2 gene resulted in the development of a UC-like disease in mice,[4] while an interleukin 10 (IL10) 'knockout' mouse developed a chronic enterocolitis.[5] The immunological perturbation found in the IL2-deficient animal strongly mirrors that found in UC with high numbers of activated T and B cells in the colonic mucosa, a reduction in $\gamma\delta$TCR+ cells, increased IgG1 and

© 1994 Rapid Communications of Oxford Ltd autoantibody levels, and the aberrant expression of MHC class 2 molecules.[11–15] No pathogenic agent could be isolated from these animals and the disease did not develop in those kept under germ-free conditions, thus implying that the inflammatory responses were set off by either non-pathogenic microbial flora or other luminal antigens.

The IL10 mutant mice developed a chronic enterocolitis involving the entire intestine, but with duodenum, proximal jejunum, and proximal colon most severely affected. However, there was normal B and T cell development together with a normal antibody response to various protein antigens. Analysis of the immune response to a standard nematode infection, however, showed a very much higher $\gamma$-interferon ($\gamma$-IFN) response in mutants compared with controls, thus indicating that although IL10 is not required for the Th2 response, it plays an important role in controlling the Th1 response.[16] As with the IL2 model, no pathogen could be isolated from diseased mice, and those kept under strict specific pathogen-free conditions suffered only a limited colitis affecting the proximal colon. Other immunoregulatory abnormalities of interest to IBD included increased class 2 expression in the colon, and a 20-fold increase in tumour necrosis factor $\alpha$ (TNF$\alpha$) and interleukin 6 (IL6) production by spleen cells from mutant mice activated with lipopolysaccharide (LPS) compared with controls.

The implications from these studies are that both Th1 and Th2 subsets are essential in maintaining immunological tolerance within the intestinal mucosa.

### Cytokines as Measures of Disease Activity

There is now good evidence to suggest that lymphocytes and macrophages within the inflammatory infiltrate of both UC and CD are in a state of increased activation. They may express a number of activation-associated antigens including the 4F2 antigen,[9] the transferrin receptor,[10] and the interleukin 2 receptor (IL2R),[10,17] all examples of early antigens. Activated immune cells are capable of producing a number of different cytokines[18] which may then contribute to the inflammatory response. This may result in resolution or in continuing tissue damage, the latter creating an environment for the development of chronic disease. Therefore, careful analysis of these cytokines may be useful in establishing the nature and activity of the disease, and in determining whether they have a role in pathogenesis.

A number of different techniques have been used to measure cytokines both in the peripheral blood (PB) and at the mucosal level in patients with inflammatory bowel disease (IBD). These have included radio-immunoassays, ELISAs and bioassays (using specific cytokine-dependent cell lines)[19–21] for estimation of protein levels, and either Northern blots[22] or reverse transcription-PCR (polymerase chain reaction)[23] for cytokine message. In addition, some groups have looked at isolated cells taken from resection specimens, whilst others have used pinch biopsies obtained at colonoscopy. The differences in approach to investigation of these diseases has naturally led to difficulties in comparing results.

Interleukin 1 (IL1), IL6 and TNF$\alpha$ are often referred to as monokines as they are predominantly secreted by activated monocytes. They have a wide range of biological functions.[24–26] Interleukin 8 is a potent neutrophil chemoattractant and is also produced by activated monocytes.[27] Increased concentrations of all four of these molecules have been found in active UC and CD, implying that they have a role in the development of the inflammatory mucosal lesion.

IL1 is a potent activator of both T and B cells, it induces synthesis of acute phase proteins (via IL6) and acts as an endogenous pyrogen. Biopsies taken from patients with active CD and UC showed higher levels of IL1 compared with healthy controls, using a bioassay. In addition, the increased production could be inhibited in a dose-dependent fashion by prednisolone.[28] However, the use of biopsies in tissue culture followed by homogenization as in this study gives an absolute value for the cytokine and not a 'per cell' estimate. In addition, it does not establish the source of this powerful mediator. Other studies, however, have tried to address some of these criticisms. Isolated mucosal mononuclear cells taken from active IBD showed significantly greater IL1$\beta$ production for both CD and UC when compared with cells taken from normal bowel.[29] This increase appeared to be on a per cell basis as there was no significant difference in the percentage of macrophages present in the isolated cell population from inflamed mucosa (median 14.5%) compared with normal mucosa (median 13%). When the mononuclear cell population was depleted of macrophages, the IL1$\beta$ activity was appreciably reduced.

In more recent studies, further attempts have been made to localize the site of IL1 production. Using the isolated LPMC model, significantly greater levels of both IL1$\alpha$ and IL1$\beta$ were found in IBD patients at the mRNA and protein levels. Enterocytes isolated from the same resection specimens showed no IL1 activity.[30] *In situ* hybridization studies in the human shows message for IL1$\beta$ in subepithelial macrophages,[31] but in the rat acetic acid model of colitis the signal is found predominantly in enterocytes.[32]

These results provide good evidence to suggest that IL1$\beta$ production is increased in active CD and UC. The main, if not the only source, is likely to be tissue macrophages infiltrating from the PB.

IL6 is another pleiotropic cytokine which can be secreted by a number of cell types including activated T cells, B cells, fibroblasts, endothelium and mesangial cells,[33] as well as monocytes. It promotes terminal differentiation of B cells into antibody-producing plasma cells,[34] induction of the acute phase response by hepatocytes[35] and is an important co-stimulatory molecule for T cells.[36] Its role has been investigated recently in various chronic inflammatory disorders including rheumatoid arthritis,[37] Kawasaki disease[38] and in IBD.[39] It has also been assessed as an early marker of the acute phase response in patients with severe burns, sepsis and acute pancreatitis.[40–42]

Serum and mucosal concentrations of IL6 are significantly higher in active CD and UC, compared with inactive disease and normal controls.[39] The positive rates in the serum ELISA assay were far higher for active CD (18/20 = 90%) compared with active UC (14/34 = 41.2%). Measurement of plasma IL6 supported this difference with the median value for active CD (32.5 pg/ml) being significantly higher than active UC (12.5 pg/ml).[43] Correlation with other markers of disease activity, however, is variable. Of those studies that have applied statistics to their results, good correlation with CDAI, ESR, CRP and platelet count was only found in those patients with mixed small and large bowel disease.[43] In another recent study, no correlation was found between IL6 and clinical indices. However, all the acute phase proteins correlated with these indices.[44] An explanation for these results may be the very short half-life of IL6 in the PB (<5 min), when compared with serum proteins (several days). In addition, patients with active IBD are likely to be on steroids, which have been shown to suppress IL6 synthesis by mononuclear cells *in vitro*.[45] Finally, the IL6 produced in the mucosa may be rapidly cleared as it passes through the liver, thus giving rise to lower values in the peripheral circulation.

Further detailed investigation of IL6 protein and mRNA has yielded interesting results. In a study of mRNA levels using PCR, IL6 was found to be significantly higher in active disease.[46] Using the isolated LPMC model, similar results were recorded but in addition, the amount of IL6 protein secreted by cells from uninvolved UC mucosa was significantly higher than that of CD.[19] This difference was not seen for TNF$\alpha$ or IL1$\beta$. When the LPMC were depleted of macrophages, there was a marked decrease in TNF$\alpha$ secretion, but only a slight decrease in IL1$\beta$, and no change in IL6. These depletion studies are supported by immunohistochemistry which shows plenty of IL6 in mucosal T and B cells, as well as in macrophages.[46]

There is no doubt that higher concentrations of IL6 are found in active IBD, contributing to inflammation, antibody production and the acute phase response. But why are plasma and serum concentrations significantly higher in CD if large amounts of this cytokine are being produced by uninvolved UC mucosa and not CD mucosa? There are two possible explanations. Firstly, the isolation technique may push the cells into a heightened state of activation, and/or secondly, there may be a greater contribution of IL6 from PB cells in CD.

Apart from its role in cachexia and endotoxic shock, TNF$\alpha$ has a number of important immunomodulatory effects including increased expression of adhesion molecules,[47,48] activation and degranulation of granulocytes and macrophages,[49,50] increases in endothelial and epithelial permeability, and enhanced epithelial cell proliferation.[51] Unlike the other monokines, TNF$\alpha$ secretion remains more tightly controlled in active IBD. Although concentrations were significantly higher in active disease compared with both inactive and normal control groups in the LPMC model,[19] spontaneous secretion was far lower than IL1$\beta$ and IL6, and the response to stimulation with poke weed mitogen (PWM) far greater. In studies of TNF$\alpha$ mRNA, no significant differences have been found between disease and control groups.[46,52] *In situ* hybridization localized message to macrophages deep in the lamina propria,[31] which correlates well with macrophage depletion studies (see above). Taken together, these results suggest that there is tight control of TNF$\alpha$ expression within the intestinal mucosa at the translational stage. The differences in expression compared with IL1 and IL6, and the overlap in immunological effects, make it tempting to suggest that this cytokine plays a far less prominent role in IBD than its fellow monokines. Further parallel studies of message and protein will be essential to confirm or refute this.

Preliminary reports of increased IL8 expression in IBD suggest that it is specific for active UC, at both mRNA[52] and protein levels.[53] However, the method of quantification used in both these studies was inadequate and a definitive study is awaited. A rise in IL8 within the mucosa would be expected in view of nature of the infiltrate, as well as the ability of other cytokines such as IL1$\beta$ and TNF to stimulate secretion of IL8 by various cell types.[54]

The colony-stimulating factors (CSF) form a separate group of cytokines which play an important role in both granulocyte and macrophage proliferation, maturation and activation.[55] For this reason and presumably because a large part of the inflammatory cell infiltrate is made up of these cells, their role in IBD has been investigated using the isolated LPMC model.[56] Cells taken from inflamed mucosa produced significantly more CSF, particularly of the macrophage-derived G-CSF, when compared with cells isolated from normal bowel. In contrast, levels of interleukin 3 (IL3) and GM-CSF were decreased compared with controls. The results were similar for both constitutive expression and following PHA stimulation. They applied to both forms of IBD, and were confirmed by slot-blot analysis of the respective mRNAs. Distribution of cell types within the LPMC population was assessed by cytospins and fluorescence analysis. There were no significant differences between disease and control groups, apart from the number of activated T cells, which as expected, was much higher in the IBD group (using IL2R and TFR expression).

The increased concentration of G-CSF correlates with the greater number of activated immune cells in diseased mucosa. It is likely to contribute to neutrophil and macrophage chemotaxis and activation, and thus perpetuate mucosal inflammation and damage. Therefore, it may represent another marker of disease activity but is unlikely to be related to disease pathogenesis. The differences observed between G-CSF, GM-CSF and IL3 production may simply reflect the cell types being recruited into the mucosa from PB, and subsequently changes in the activity of other immunoregulatory cytokines that control CSF production such as IL2.

### Cytokines and Disease Pathogenesis: The Role of Th1 and Th2 Subsets

The division of CD4+ T cells into Th1 and Th2 subsets depending on their lymphokine secretion patterns was first described in a murine model.[57] Subsequently, a similar model has been considered for the human CD4+ T cell population, with the two subsets having reciprocal effects on each other.[58] Supporting evidence for this *in vivo* comes from the study of infections which generate a spectrum of immune responses in their hosts. These include leprosy and leishmaniasis.[59,60] Analysis of cytokine profiles in patients with different forms of the same disease revealed that each subset was associated with a specific immune response, thus generating its own disease phenotype. Therefore, in patients with tuberculoid leprosy, the Th1 subset (IL2 and γ-IFN) predominates giving rise to a cellular immune response and eradication of the organism. In the lepromatous form, the Th2 subset (IL4, IL5 and IL10) predominates stimulating a humoral response and persistence of infection.[59] However, predominance of Th2 cells is not always associated with susceptibility. In certain situations, a dominant Th2 response is required to keep an inappropriate and potentially immunopathologic Th1 response in check.[61]

The role of these immunomodulatory cytokines in IBD has generated much interest recently, particularly with the development of spontaneous intestinal inflammation in the IL2 and IL10 'knockout mice' (see above). At present, there is some disagreement over the concentrations of IL2 in active IBD with both increases and decreases being recorded. One obvious explanation for this is the difference in materials and methods used. IL2 has been extensively studied in the isolated LPMC model,[21,22,62,63] with consistently reduced levels found in cells taken from inflamed mucosa compared with normal or uninvolved bowel. The low IL2 protein and mRNA levels are attributed to a loss of CD4+ IL2-producing intestinal T cells.[63] There is one exception to these findings using the same model, with no significant differences in IL2 production between any of the groups.[64] In all these studies, the authors insist that the type of therapy (e.g. corticosteroids) does not appear to influence IL2 levels. However, it is well-established that corticosteroids inhibit IL2 mRNA expression[65] and 5-aminosalicylic acid also appears to inhibit secretion of this cytokine at concentrations well within the faecal fluid therapeutic range of 3 to 20 mM.[64]

The study of IL2R expression[66] suggests a reciprocal relationship with its ligand. Receptor levels are increased in CD and unchanged in UC, whereas IL2 levels measured by the same group have always been reduced for both diseases. Most of the receptor activity is within the T cell compartment, and coexpression of $\alpha$ and $\beta$ chains is likely in view of the large amounts of mRNA found for both. Whether the IL2 deficiency is a primary phenomenon, is related to the cell isolation technique, or to greater binding and internalization by a larger number of receptors is yet to be established.

Using a mixed cell population within colonoscopic biopsies, and a quantitative PCR technique, IL2 mRNA expression was found to be significantly higher in active CD compared with active UC and normal controls.[23] The presence of an increased number of immune cells within the IBD biopsies was not controlled for; analysis of CD3$\delta$ mRNA by PCR may have been a suitable internal standard to correct for this. A similar criticism may be levelled at an earlier study of IL2 protein using sonicated biopsies.[67]

The other major component of the Th1 subset is γ-IFN. This cytokine has a central role in controlling development of the immune response, with induction of a number of phagocyte and lymphocyte functions[68] and potent effects on the expression of cell surface antigens including MHC

class 2.[69] Further evidence for this comes from the multiple immunological defects seen in mice with a disrupted γ-IFN gene.[70] In isolated LPMC, γ-IFN concentrations were found to be significantly reduced in cells taken from inflamed sites compared with areas of uninflamed mucosa and normal controls.[71] No γ-IFN was detected in unstimulated cultures, but it could be induced equally by both IL2 and PHA (1%). Depletion of the adherent cell population generally resulted in a decrease in the amount of γ-IFN detected. Analysis of T cells from each group showed no significant differences either in the absolute number of CD3+ cells or in the subsets. Clinical disease activity and corticosteroid treatment was said not to correlate with γ-IFN concentrations.

Two other studies have found the opposite result, with γ-IFN concentrations being higher in active CD compared with controls.[72,73] UC was not assessed. However, on investigating the kinetics of γ-IFN production after stimulation of LPMC, Fais *et al.*[72] found that the CD LPMC peaked at 24 h and then plateaued, whilst control cells produced increasing amounts up to 72 h, when the experiment was terminated. This observation, when taken together with the other studies, suggests that T cells within the inflamed mucosa may already have their Th1 cytokine genes 'turned on' to near maximal levels, and hence further increases in expression are limited or impossible. The reduced concentrations found in the first study cited could also be due to alterations in cell type in the mucosa, a defect in macrophage-T cell interaction or secondary to reduced IL2 levels.[74] However, it is difficult to reconcile these results with the obvious increase in class 2 expression in inflamed colon, the latter thought to be predominantly mediated by γ-IFN.

An explanation for the limited or reduced Th1 response in active IBD may be found by studying the activity of the Th2 subset, as has been done in infectious diseases (see above). Work in this area is limited, and has been presented in abstract form only. IL4 is essential for the development of a full Th2 response.[75] It has a number of functions including activation of B cells,[76] control of Ig isotype switching,[77] and inhibitory effects on macrophages.[78] Two studies[79,80] using the LPMC model show a reduced inhibitory response to recombinant IL4 in IBD. In one, γ-IFN and IL2R mRNA were measured as markers of Th1 activity, and in the other, macrophage activity was assessed using mannose receptor expression and superoxide generation. Assessment of IL4 mRNA and protein at the mucosal level has not produced any consistent results as yet.

Interleukin 10 has powerful inhibitory effects on synthesis of Th1 cytokines,[16] and can suppress macrophage activation *in vitro*.[81] In view of the extensive inflammation with T cell and macrophage activation seen in IBD, investigation of its role is essential but so far limited. In a small study using colonoscopic biopsies and the RT-PCR method, IL10 mRNA was found to be higher in active UC compared with active CD.[82] Taken together with the IL2 data from the same author,[23] a theory of CD being a Th1-driven disease and UC a Th2-driven disease has been put forward. Using similar methods, however, another study has found IL10 equally elevated in active CD and UC (unpublished observations). At present, no major conclusions can be made on the role of the Th2 cytokines in IBD.

### Conclusions

Cytokines undoubtedly play an important role in the development and persistence of the inflammatory lesions seen in CD and UC. There is now good evidence to suggest that both IL1 and IL6 are significantly elevated in active disease, and may be used as markers of disease activity at the mucosal level. The use of serum or plasma IL6 as a marker of activity, however, is limited in view of its very short half-life. Control of TNFα release appears to be much tighter when compared with the other monokines, and far more dependent on activated macrophages as its source. Its contribution to the inflammatory lesion is probably less important in IBD.

The role of the immunoregulatory cytokines within the Th1 and Th2 subsets is complicated by conflicting results, and a lack of data. The lower concentrations of IL2 and γ-IFN in active disease are consistent with the apparent loss of immunological tolerance seen in the IL2 knockout model. Further study of differences between involved and uninvolved mucosa in the same patient may help to establish whether the Th1 deficiency is merely a reflection of infiltration of new, naive cells or a predominance of primed, 'exhausted' cells. The concept of a full Th1/Th2 profile in each patient is attractive and has already yielded useful information regarding the nature of the immune response in the field of infectious disease. This approach may not only elucidate whether these cytokines have a primary role in the pathogenesis of IBD, but can also help to establish whether there are fundamental differences in the immune response in CD and UC.

### References

1. Paul WE. Pleiotropy and redundancy: T cell derived lymphokines in the immune response. *Cell* 1989; **57**: 521–574.
2. Podolsky DK. Inflammatory bowel disease. *New Engl J Med* 1991; **325**: 928–937.
3. Hammer RE, Maika SD, Richardson JA, Tang J-P, Tanrog JD. Spontaneous inflammatory disease in transgenic rats expressing HLA-B27 and human β2M: an animal model of HLA-B27-associated human disorders. *Cell* 1990; **63**: 1099–1112.

4. Sadlack B, Merz H, Schoile H, Schimpl A, Feller AC, Horak I. Ulcerative colitis-like disease in mice with a disrupted interleukin-2 gene. *Cell* 1993; **75**: 253–261.
5. Kuhn R, Lohler J, Rennick D, Rajewsky K, Muller W. Interleukin-10-deficient mice develop chronic enterocolitis. *Cell* 1993; **75**: 263–274.
6. Mombaerts P, Mizoguchi E, Grusby MJ, Glimcher LH, Bhan AK, Tonegawa S. Spontaneous development of inflammatory bowel disease in T cell receptor mutant mice. *Cell* 1993; **75**: 275–282.
7. Stober W. Animal models of inflammatory bowel disease—an overview. *Dig Dis Sci* 1985; **30**: 3S–10S.
8. Gurbindo C, Russo P, Sabbah S, Lohoves M-J, Seidman E. Interleukin 2 activity of colonic lamina propria mononuclear cells in a rat model of experimental colitis. *Gastroenterology* 1993; **104**: 964–972.
9. Pallone F, Fais S, Squarcia O, Biancone L, Pozzilli P, Boivirant M. Activation of peripheral blood and intestinal lymphocytes in Crohn's disease. *In vivo* state of activation and *in vitro* response to stimulation as defined by the expression of early activation antigens. *Gut* 1987; **28**: 745–753.
10. Schreiber S, MacDermott RP, Raedler A, Pinnau R, Bertovich MJ, Nash GS. Increased activation of isolated lamina propria mononuclear cells in inflammatory bowel disease. *Gastroenterology* 1991; **101**: 1020–1030.
11. MacDermott RP, Nash GS, Bertovich MJ, Seiden MV, Bragdon MJ, Beale MG. Alterations of IgM, IgG, and IgA synthesis and secretion by peripheral blood and intestinal mononuclear cells from patients with ulcerative colitis and Crohn's disease. *Gastroenterology* 1981; **81**: 844–852.
12. Kobayashi K, Asakura H, Hamada Y. T lymphocyte subpopulations and immunoglobulin-containing cells in the colonic mucosa of ulcerative colitis: morphometric and immunohistochemical study. *J Clin Lab Immunol* 1988; **25**: 63–68.
13. Hibi T, Ohara M, Toda K, *et al. In vitro* anticolon antibody production by mucosal or peripheral blood lymphocytes from patients with ulcerative colitis. *Gut* 1990; **31**: 1371–1376.
14. Fukushima K, Masuda T, Ohtani H, *et al.* Immunohistochemical characterization, distribution, and ultrastructure of lymphocytes bearing T cell receptor $\gamma/\delta$ in inflammatory bowel disease. *Gastroenterology* 1991; **101**: 670–678.
15. Mayer L, Eisenhardt D, Salomon P, Bauer W, Plous R, Piccinini L. Expression of class 2 molecules on intestinal epithelial cells in humans. *Gastroenterology* 1991; **100**: 3–12.
16. Fiorentino DF, Bond MW, Mossmann TR. Two types of mouse T helper cell. IV. Th2 clones secrete a factor that inhibits cytokine production by Th1 clones. *J Exp Med* 1989; **170**: 2081–2095.
17. Choy MY, Walker-Smith JA, Williams CB, MacDonald TT. Differential expression of CD25 (interleukin-2 receptor) on lamina propria T cells and macrophages in the intestinal lesions in Crohn's disease and ulcerative colitis. *Gut* 1990; **321**: 1365–1370.
18. Balkmill FR, Burke F. The cytokine network. *Immunol Today* 1989; **10**: 299–304.
19. Reinecker H, Steffen M, Witthoeft T, Pflueger I, Schreiber S, MacDermott R-P, Raedler A. Enhanced secretion of tumour necrosis factor-alpha, IL-6, and IL-1$\beta$ by isolated lamina propria mononuclear cells from patients with ulcerative colitis and Crohn's disease. *Clin Exp Immunol* 1993; **94**: 174–181.
20. Gillis S, Smith KA. Long term culture of tumour-specific cytotoxic T cells. *Nature* 1977; **268**: 154–156.
21. Fiocchi C, Hilfiker ML, Youngman KR, Doerder NC, Finke JH. Interleukin 2 activity of human intestinal mucosa mononuclear cells. Decreased levels in inflammatory bowel disease. *Gastroenterology* 1984; **86**: 734–742.
22. Matsuura T, West G, Youngman KR, Klein JS, Fiocchi C. Immune activation genes in inflammatory bowel disease. *Gastroenterology* 1993; **104**: 448–458.
23. Mullin GE, Lazenby AJ, Harris ML, Bayless TM, James SP. Increased interleukin-2 messenger RNA in the intestinal mucosal lesions of Crohn's disease but not ulcerative colitis. *Gastroenterology* 1992; **102**: 1620–1627.
24. Dinarello C. Interleukin 1 and interleukin 1 antagonism. *Blood* 1991; **77**: 1627–1652.
25. Hirano T, Akira S, Taga T, Kishimoto T. Biological and clinical aspects of interleukin 6. *Immunol Today* 1990; **11**: 443–449.
26. Vilcek J, Lee T. Tumour necrosis factor. New insights into the molecular mechanisms of its actions. *J Biol Chem* 1991; **266**: 7313–7316.
27. Schroder JJ, Mrowietz U, Morita E, Christophers E. Purification and partial biochemical characterization of a human monocyte-derived, neutrophil-activating peptide that lacks interleukin 1 activity. *J Immunol* 1987; **139**: 3473–3483.
28. Ligumsky M, Simon PL, Karmeli F, Rachmilewitz D. Role of interleukin 1 in inflammatory bowel disease—enhanced production during active disease. *Gut* 1990; **31**: 686–689.
29. Mahida YR, Wu K, Jewell DP. Enhanced production of interleukin-1$\beta$ by mononuclear cells isolated from mucosa with active ulcerative colitis or Crohn's disease. *Gut* 1989; **30**: 835–838.
30. Youngman KR, Simon PL, West GA, Cominelli F, Rachmilewitz D, Klein JS, Fiocchi C. Localization of intestinal interleukin 1 activity and protein and gene expression to lamina propria cells. *Gastroenterology* 1993; **104**: 749–758.
31. Capello M, Keshav S, Prince C, Jewell DP, Gordon S. Detection of mRNAs for macrophage products in inflammatory bowel disease by *in situ* hybridization. *Gut* 1992; **33**: 1214–1219.
32. Radema SA, Van Deventer SJH, Cerami A. Interleukin 1$\beta$ is expressed predominantly by enterocytes in experimental colitis. *Gastroenterology* 1991; **100**: 1180–1186.
33. Van Snick J. Interleukin 6: an overview. *Ann Rev Immunol* 1990; **8**: 253–278.
34. Raynal MC, Lin Z, Hirano T, Mayer L, Kishimoto T, Chen-Kiang S. Interleukin 6 induces secretion of IgG1 by coordinated transcriptional activation and differential mRNA accumulation. *Proc Natl Acad Sci USA* 1989; **86**: 9024–9028.
35. Gauldie J, Richards C, Harmish D, Landsdorp P, Baumann H. Interferon $\beta$2/B-cell stimulatory factor type 2 shares identity with monocyte-derived hepatocyte-stimulating factor and regulates the major acute phase protein response in liver cells. *Proc Natl Acad Sci USA* 1987; **84**: 7251–7255.
36. Okada M, Kitahara M, Kishimoto S, Matsuda T, Hirano T, Kishimoto T. IL6/BSF-2 functions as a killer helper factor in the *in vitro* induction of cytotoxic T cells. *J Immunol* 1988; **141**: 1543–1549.
37. Houssiau FA, Devogelaer JP, Damme JV, *et al.* Interleukin-6 in synovial fluid and serum of patients with rheumatoid arthritis and other inflammatory arthritides. *Arth Rheum* 1988; **31**: 784–788.
38. Ueno Y, Takano N, Kanegane H, *et al.* The acute phase nature of interleukin 6. Studies in Kawasaki disease and other febrile illnesses. *Clin Exp Immunol* 1989; **76**: 337–342.
39. Mitsuyama K, Sata M, Tanikawa K. Significance of interleukin-6 in patients with inflammatory bowel disease. *Gastroenterologia Japonica* 1991; **26**: 20–28.
40. Nijsten MW, DeGroot ER, TenDuis HJ, Klesen HJ, Hack CE, Aarden LA. Serum levels of interleukin 6 and acute phase responses (letter). *Lancet* 1987; **ii**: 921.
41. Waage A, Brandtzaeg P, Halstensen A, Kievulf P, Espevik T. The complex pattern of cytokines in serum from patients with meningococcal septic shock. Association between interleukin-6, interleukin-1, and fatal outcome. *J Exp Med* 1989; **169**: 333–338.
42. Leser HG, Gross V, Scheibenbogen C, *et al.* Elevation of serum interleukin-6 concentration precedes acute-phase response and reflects severity in acute pancreatitis. *Gastroenterology* 1991; **101**: 782–785.
43. Lobo AJ, Evans SW, Jones SC, *et al.* Plasma interleukin-6 in inflammatory bowel disease. *Eur J Gastro Hep* 1992; **4**: 367–372.
44. Gross V, Andus T, Caesar J, Roth M, Schölmerich J. Evidence for continuous stimulation of interleukin-6 production in Crohn's disease. *Gastroenterology* 1992; **102**: 514–519.
45. Woloski BM, Smith EM, Meyer WJ, Fuller GM, Blalozk JE. Corticotropin-releasing activity of monokines. *Science* 1985; **230**: 1035–1037.
46. Stevens C, Walz G, Singaram C, *et al.* Tumour necrosis factor-$\alpha$, interleukin-1$\beta$, and interleukin-6 expression in inflammatory bowel disease. *Dig Dis Sci* 1992; **37**: 818–826.
47. Bevilacqua MP, Pober JS, Mendrik DL, Cotran RS, Gimbrone MA Jr. Identification of an inducible endothelial leucocyte adhesion molecule. *Proc Natl Acad Sci USA* 1987; **84**: 9238–9242.
48. Pober JS, Lapierre LA, Stolpen AH, Brock TA, Springer TA. Activation of cultured human endothelial cells by recombinant lymphotoxin: comparison with tumour necrosis factor and interleukin 1 species. *J Immunol* 1987; **138**: 3319–3324.
49. Klebanoff SJ, Vadas MA, Harlan JM, *et al.* Stimulation of neutrophils by tumour necrosis factor. *J Immunol* 1986; **136**: 4220–4225.
50. Hoffman M, Weinberg JB. Tumour necrosis factor-$\alpha$ induces increased hydrogen peroxide production and Fc receptor expression, but not increased 1a antigen expression by peritoneal macrophages. *J Leukocyte Biol* 1987; **42**: 704–707.
51. Vidrich A, Anton P, Shanahan F. Cytokines modulate the growth of normal colonic epithelia. *Gastroenterology* 1992; **102**: A701.
52. Isaacs KL, Balfour Sartor R, Haskill S. Cytokine mesanger RNA profiles in inflammatory bowel disease detected by polymerase chain reaction amplification. *Gastroenterology* 1992; **103**: 1587–1595.
53. Mahida YR, Coska M, Effenberger F, Kurlak L, Lindley I, Hawkey CJ. Enhanced synthesis of neutrophil-activating peptide-1/interleukin-8 in active ulcerative colitis. *Clin Sci* 1992; **82**: 273–275.
54. Matsushima K, Morishita Y, Yoshimura T, *et al.* Molecular cloning of a human monocyte-derived neutrophil chemotactic factor (MDNCF) and the induction of MDNCF mRNA by interleukin-1 and tumour necrosis factor. *J Exp Med* 1988; **167**: 1883–1893.
55. Clark SC, Kamen R. The human haemopoietic colony-stimulating factors. *Science* 1987; **236**: 1229–1237.
56. Pullman WE, Elsbury S, Kobayashi M, Hapel AJ, Doe WF. Enhanced mucosal cytokine production in inflammatory bowel disease. *Gastroenterology* 1992; **102**: 529–537.
57. Mosmann TR, Coffman RL. Th1 and Th2 cells: different patterns of lymphokine secretion lead to different functional properties. *Ann Rev Immunol* 1989; **7**: 145–173.
58. Maggi E, Parronchi P, Maretti R, *et al.* Reciprocal regulatory effects of IFN-gamma and IL-4 on the *in vitro* development of human Th1 and Th2 clones. *J Immunol* 1992; **148**: 2142–2147.
59. Yamamura M, Uyemura K, Deans RJ, *et al.* Defining protective responses to pathogens: cytokine profiles in leprosy lesions. *Science* 1991; **254**: 277–279.
60. Caceres-Dittmar G, Tapia FJ, Sanchez MA, *et al.* Determination of the cytokine profile in American cutaneous leishmaniasis using the polymerase chain reaction. *Clin Exp Immunol* 1993; **91**: 500–505.
61. Urban JF, Madden KB, Svetic A, *et al.* The importance of the Th2 cytokines in protective immunity to nematodes. *Immunol Rev* 1992; **127**: 205–220.
62. Kusugami K, Youngman KR, West GA, Fiocchi C. Intestinal immune reactivity to interleukin 2 differs among Crohn's disease, ulcerative colitis, and controls. *Gastroenterology* 1989; **97**; 1–9.

63. Kusugami K, Matsuura T, West GA, Youngman KR, Rachmilewitz D, Fiocchi C. Loss of interleukin-2-producing CD4+ T cells in inflammatory bowel disease. *Gastroenterology* 1991; **101**: 1594–1605.
64. Pullman WE, Doe WF. IL-2 production by intestinal lamina propria cells in normal inflamed and cancer-bearing colons. *Clin Exp Immunol* 1992; **88**: 132–137.
65. Arya SK, Wong-Staal F, Gallo R. Dexamethasone-mediated inhibition of human T cell growth factor and gamma-interferon messenger RNA. *J Immunol* 1984; **133**: 273–276.
66. Matsuura T, West GA, Klein JS, Ferraris L, Fiocchi C. Soluble interleukin 2 and CD8 and CD4 receptors in inflammatory bowel disease. *Gastroenterology* 1992; **102**: 2006–2014.
67. Brynskov J, Tvede N, Andersen CB, Vilien M. Increased concentrations of interleukin-1$\beta$, interleukin-2, and soluble interleukin-2 receptors in endoscopical mucosal biopsy specimens with active inflammatory bowel disease. *Gut* 1992; **33**: 55–58.
68. De Mayer, E, De Mayer-Guignard J. Production of INF-$\gamma$ by T cells and modulation of T cells, B cell and NK activity by interferons. In: De Mayer E, De Mayer-Guignard J, eds. *Interferons and Other Regulatory Cytokines*. New York: John Wiley, 1988; 221–273.
69. Capobianchi MR, Ameglio F, Tosi R, Dolei A. Difference in the expression and release of DR, BR and DQ molecules in human cells treated with recombinant interferon gamma: comparison to other interferons. *Hum Immunol* 1985; **13**: 1–5.
70. Dalton DK, Pitts-Meeks S, Keshav S, Figari IS, Bradley A, Stewart TA. Multiple defects of immune cell function in mice with disrupted interferon-$\gamma$ genes. *Science* 1993; **259**: 1739–1742.
71. Lieberman BY, Fiocchi C, Youngman KR, Sapatnekar WK, Proffitt MR. Interferon $\gamma$ production by human intestinal mucosal mononuclear cells. Decreased levels in inflammatory bowel disease. *Dig Dis Sci* 1988; **33**: 1297–1304.
72. Fais S, Capobianchi MR, Pallone F, Di Marco P, Boirivant M, Dianzani M, Torsoli A. Spontaneous release of interferon $\gamma$ by intestinal lamina propria lymphocytes in Crohn's disease. Kinetics of *in vitro* response to interferon $\gamma$ inducers. *Gut* 1991; **32**: 403–407.
73. Sasaki T, Hiwatashi N, Yamazaki H, Noguchi M, Toyota T. The role of inteferon $\gamma$ in the pathogenesis of Crohn's disease. *Gastroenterologica Japonica* 1992; **27**: 29–36.
74. Kasahara T, Hooks JJ, Dougherty SF, Oppenheim JJ. Interleukin 2-mediated immune interferon (IFN-$\gamma$) production by human T cells and T cell subsets. *J Immunol* 1983; **130**: 1784–1789.
75. Kopf M, Le Gros G, Bachmann M, Lamers MC, Bluethmann H, Kohler G. Disruption of the murine IL-4 gene blocks Th2 cytokine responses. *Nature* 1993; **362**: 245–247.
76. Yokota T, Otsuka T, Mosmann T, *et al.* Isolation and characterization of a human cDNA clone, homologous to mouse B-cell stimulatory factor 1, that expresses B-cell- and T-cell-stimulating activities. *Proc Natl Acad Sci USA* 1986; **83**: 5894–5898.
77. Snapper CM, Finkelman FD, Paul WE. Differential regulation of IgG1 and IgE synthesis by interleukin 4. *J Exp Med* 1988; **167**: 183–196.
78. Essner R, Rhoades K, McBride WH, Morton DL, Economou JS. IL-4 down-regulates IL-1 and TNF gene expression in human monocytes. *J Immunol* 1989; **142**: 3857–3865.
79. Matsuura T, West GA, Klein JS, Levine AD, Kusugami K, Morise K, Fiocchi C. Immune activation gene products are resistant to IL-4 inhibitory activity in Crohn's disease (CD). *Gastroenterology* 1993; **104**: A739.
80. Schreiber S, Blum JS, Howaldt S, Berghaus D, Baldassano RN, Raedler A. Deactivation of intestinal macrophages and induction of mannose receptor expression by interleukin 4: defective regulation in IBD. *Gastroenterology* 1993; **104**: A776.
81. Fiorentino DF, Zlotnik A, Mosmann TR, Howard M, O'Garra A. IL-10 inhibits cytokine production by activated macrophages. *J Immunol* 1991; **147**: 3815–3822.
82. Mullin GE, Vezza FR, Sampat A, *et al.* Abnormal IL-10 mRNA production in the intestinal mucosal lesions of inflammatory bowel disease. *Gastroenterology* 1993; **104**: A751.

**Received and accepted 30 November 1993**

## References

[PDF_00665] Balkwill F. R., Burke F. (1989). The cytokine network.. Immunol Today.

[PDF_00748] Bevilacqua M. P., Pober J. S., Mendrick D. L., Cotran R. S., Gimbrone M. A. (1987). Identification of an inducible endothelial-leukocyte adhesion molecule.. Proc Natl Acad Sci U S A.

[PDF_00808] Brynskov J., Tvede N., Andersen C. B., Vilien M. (1992). Increased concentrations of interleukin 1 beta, interleukin-2, and soluble interleukin-2 receptors in endoscopical mucosal biopsy specimens with active inflammatory bowel disease.. Gut.

[PDF_00816] Capobianchi M. R., Ameglio F., Tosi R., Dolei A. (1985). Differences in the expression and release of DR, BR, and DQ molecules in human cells treated with recombinant interferon gamma: comparison to other interferons.. Hum Immunol.

[PDF_00702] Cappello M., Keshav S., Prince C., Jewell D. P., Gordon S. (1992). Detection of mRNAs for macrophage products in inflammatory bowel disease by in situ hybridisation.. Gut.

[PDF_00661] Choy M. Y., Walker-Smith J. A., Williams C. B., MacDonald T. T. (1990). Differential expression of CD25 (interleukin-2 receptor) on lamina propria T cells and macrophages in the intestinal lesions in Crohn's disease and ulcerative colitis.. Gut.

[PDF_00773] Clark S. C., Kamen R. (1987). The human hematopoietic colony-stimulating factors.. Science.

[PDF_00786] Cáceres-Dittmar G., Tapia F. J., Sánchez M. A., Yamamura M., Uyemura K., Modlin R. L., Bloom B. R., Convit J. (1993). Determination of the cytokine profile in American cutaneous leishmaniasis using the polymerase chain reaction.. Clin Exp Immunol.

[PDF_00820] Dalton D. K., Pitts-Meek S., Keshav S., Figari I. S., Bradley A., Stewart T. A. (1993). Multiple defects of immune cell function in mice with disrupted interferon-gamma genes.. Science.

[PDF_00682] Dinarello C. A. (1991). Interleukin-1 and interleukin-1 antagonism.. Blood.

[PDF_00827] Fais S., Capobianchi M. R., Pallone F., Di Marco P., Boirivant M., Dianzani F., Torsoli A. (1991). Spontaneous release of interferon gamma by intestinal lamina propria lymphocytes in Crohn's disease. Kinetics of in vitro response to interferon gamma inducers.. Gut.

[PDF_00673] Fiocchi C., Hilfiker M. L., Youngman K. R., Doerder N. C., Finke J. H. (1984). Interleukin 2 activity of human intestinal mucosa mononuclear cells. Decreased levels in inflammatory bowel disease.. Gastroenterology.

[PDF_00658] Fiorentino D. F., Bond M. W., Mosmann T. R. (1989). Two types of mouse T helper cell. IV. Th2 clones secrete a factor that inhibits cytokine production by Th1 clones.. J Exp Med.

[PDF_00651] Fukushima K., Masuda T., Ohtani H., Sasaki I., Funayama Y., Matsuno S., Nagura H. (1991). Immunohistochemical characterization, distribution, and ultrastructure of lymphocytes bearing T-cell receptor gamma/delta in inflammatory bowel disease.. Gastroenterology.

[PDF_00713] Gauldie J., Richards C., Harnish D., Lansdorp P., Baumann H. (1987). Interferon beta 2/B-cell stimulatory factor type 2 shares identity with monocyte-derived hepatocyte-stimulating factor and regulates the major acute phase protein response in liver cells.. Proc Natl Acad Sci U S A.

[PDF_00667] Gillis S., Smith K. A. (1977). Long term culture of tumour-specific cytotoxic T cells.. Nature.

[PDF_00740] Gross V., Andus T., Caesar I., Roth M., Schölmerich J. (1992). Evidence for continuous stimulation of interleukin-6 production in Crohn's disease.. Gastroenterology.

[PDF_00630] Gurbindo C., Russo P., Sabbah S., Lohoues M. J., Seidman E. (1993). Interleukin-2 activity of colonic lamina propria mononuclear cells in a rat model of experimental colitis.. Gastroenterology.

[PDF_00614] Hammer R. E., Maika S. D., Richardson J. A., Tang J. P., Taurog J. D. (1990). Spontaneous inflammatory disease in transgenic rats expressing HLA-B27 and human beta 2m: an animal model of HLA-B27-associated human disorders.. Cell.

[PDF_00648] Hibi T., Ohara M., Toda K., Hara A., Ogata H., Iwao Y., Watanabe N., Watanabe M., Hamada Y., Kobayashi K. (1990). In vitro anticolon antibody production by mucosal or peripheral blood lymphocytes from patients with ulcerative colitis.. Gut.

[PDF_00684] Hirano T., Akira S., Taga T., Kishimoto T. (1990). Biological and clinical aspects of interleukin 6.. Immunol Today.

[PDF_00757] Hoffman M., Weinberg J. B. (1987). Tumor necrosis factor-alpha induces increased hydrogen peroxide production and Fc receptor expression, but not increased Ia antigen expression by peritoneal macrophages.. J Leukoc Biol.

[PDF_00720] Houssiau F. A., Devogelaer J. P., Van Damme J., de Deuxchaisnes C. N., Van Snick J. (1988). Interleukin-6 in synovial fluid and serum of patients with rheumatoid arthritis and other inflammatory arthritides.. Arthritis Rheum.

[PDF_00763] Isaacs K. L., Sartor R. B., Haskill S. (1992). Cytokine messenger RNA profiles in inflammatory bowel disease mucosa detected by polymerase chain reaction amplification.. Gastroenterology.

[PDF_00644] Kobayashi K., Asakura H., Hamada Y., Hibi T., Watanabe M., Yoshida T., Watanabe N., Miura S., Aiso S., Tsuchiya M. (1988). T lymphocyte subpopulations and immunoglobulin-containing cells in the colonic mucosa of ulcerative colitis; a morphometric and immunohistochemical study.. J Clin Lab Immunol.

[PDF_00834] Kopf M., Le Gros G., Bachmann M., Lamers M. C., Bluethmann H., Köhler G. (1993). Disruption of the murine IL-4 gene blocks Th2 cytokine responses.. Nature.

[PDF_00791] Kusugami K., Matsuura T., West G. A., Youngman K. R., Rachmilewitz D., Fiocchi C. (1991). Loss of interleukin-2-producing intestinal CD4+ T cells in inflammatory bowel disease.. Gastroenterology.

[PDF_00623] Kühn R., Löhler J., Rennick D., Rajewsky K., Müller W. (1993). Interleukin-10-deficient mice develop chronic enterocolitis.. Cell.

[PDF_00735] Leser H. G., Gross V., Scheibenbogen C., Heinisch A., Salm R., Lausen M., Rückauer K., Andreesen R., Farthmann E. H., Schölmerich J. (1991). Elevation of serum interleukin-6 concentration precedes acute-phase response and reflects severity in acute pancreatitis.. Gastroenterology.

[PDF_00823] Lieberman B. Y., Fiocchi C., Youngman K. R., Sapatnekar W. K., Proffitt M. R. (1988). Interferon gamma production by human intestinal mucosal mononuclear cells. Decreased levels in inflammatory bowel disease.. Dig Dis Sci.

[PDF_00692] Ligumsky M., Simon P. L., Karmeli F., Rachmilewitz D. (1990). Role of interleukin 1 in inflammatory bowel disease--enhanced production during active disease.. Gut.

[PDF_00640] MacDermott R. P., Nash G. S., Bertovich M. J., Seiden M. V., Bragdon M. J., Beale M. G. (1981). Alterations of IgM, IgG, and IgA Synthesis and secretion by peripheral blood and intestinal mononuclear cells from patients with ulcerative colitis and Crohn's disease.. Gastroenterology.

[PDF_00781] Maggi E., Parronchi P., Manetti R., Simonelli C., Piccinni M. P., Rugiu F. S., De Carli M., Ricci M., Romagnani S. (1992). Reciprocal regulatory effects of IFN-gamma and IL-4 on the in vitro development of human Th1 and Th2 clones.. J Immunol.

[PDF_00766] Mahida Y. R., Ceska M., Effenberger F., Kurlak L., Lindley I., Hawkey C. J. (1992). Enhanced synthesis of neutrophil-activating peptide-1/interleukin-8 in active ulcerative colitis.. Clin Sci (Lond).

[PDF_00695] Mahida Y. R., Wu K., Jewell D. P. (1989). Enhanced production of interleukin 1-beta by mononuclear cells isolated from mucosa with active ulcerative colitis of Crohn's disease.. Gut.

[PDF_00769] Matsushima K., Morishita K., Yoshimura T., Lavu S., Kobayashi Y., Lew W., Appella E., Kung H. F., Leonard E. J., Oppenheim J. J. (1988). Molecular cloning of a human monocyte-derived neutrophil chemotactic factor (MDNCF) and the induction of MDNCF mRNA by interleukin 1 and tumor necrosis factor.. J Exp Med.

[PDF_00802] Matsuura T., West G. A., Klein J. S., Ferraris L., Fiocchi C. (1992). Soluble interleukin 2 and CD8 and CD4 receptors in inflammatory bowel disease.. Gastroenterology.

[PDF_00676] Matsuura T., West G. A., Youngman K. R., Klein J. S., Fiocchi C. (1993). Immune activation genes in inflammatory bowel disease.. Gastroenterology.

[PDF_00655] Mayer L., Eisenhardt D., Salomon P., Bauer W., Plous R., Piccinini L. (1991). Expression of class II molecules on intestinal epithelial cells in humans. Differences between normal and inflammatory bowel disease.. Gastroenterology.

[PDF_00726] Mitsuyama K., Sata M., Tanikawa K. (1991). Significance of interleukin-6 in patients with inflammatory bowel disease.. Gastroenterol Jpn.

[PDF_00625] Mombaerts P., Mizoguchi E., Grusby M. J., Glimcher L. H., Bhan A. K., Tonegawa S. (1993). Spontaneous development of inflammatory bowel disease in T cell receptor mutant mice.. Cell.

[PDF_00778] Mosmann T. R., Coffman R. L. (1989). TH1 and TH2 cells: different patterns of lymphokine secretion lead to different functional properties.. Annu Rev Immunol.

[PDF_00679] Mullin G. E., Lazenby A. J., Harris M. L., Bayless T. M., James S. P. (1992). Increased interleukin-2 messenger RNA in the intestinal mucosal lesions of Crohn's disease but not ulcerative colitis.. Gastroenterology.

[PDF_00728] Nijsten M. W., de Groot E. R., ten Duis H. J., Klasen H. J., Hack C. E., Aarden L. A. (1987). Serum levels of interleukin-6 and acute phase responses.. Lancet.

[PDF_00717] Okada M., Kitahara M., Kishimoto S., Matsuda T., Hirano T., Kishimoto T. (1988). IL-6/BSF-2 functions as a killer helper factor in the in vitro induction of cytotoxic T cells.. J Immunol.

[PDF_00633] Pallone F., Fais S., Squarcia O., Biancone L., Pozzilli P., Boirivant M. (1987). Activation of peripheral blood and intestinal lamina propria lymphocytes in Crohn's disease. In vivo state of activation and in vitro response to stimulation as defined by the expression of early activation antigens.. Gut.

[PDF_00610] Paul W. E. (1989). Pleiotropy and redundancy: T cell-derived lymphokines in the immune response.. Cell.

[PDF_00751] Pober J. S., Lapierre L. A., Stolpen A. H., Brock T. A., Springer T. A., Fiers W., Bevilacqua M. P., Mendrick D. L., Gimbrone M. A. (1987). Activation of cultured human endothelial cells by recombinant lymphotoxin: comparison with tumor necrosis factor and interleukin 1 species.. J Immunol.

[PDF_00612] Podolsky D. K. (1991). Inflammatory bowel disease (1). N Engl J Med.

[PDF_00799] Pullman W. E., Doe W. F. (1992). IL-2 production by intestinal lamina propria cells in normal inflamed and cancer-bearing colons.. Clin Exp Immunol.

[PDF_00775] Pullman W. E., Elsbury S., Kobayashi M., Hapel A. J., Doe W. F. (1992). Enhanced mucosal cytokine production in inflammatory bowel disease.. Gastroenterology.

[PDF_00705] Radema S. A., van Deventer S. J., Cerami A. (1991). Interleukin 1 beta is expressed predominantly by enterocytes in experimental colitis.. Gastroenterology.

[PDF_00620] Sadlack B., Merz H., Schorle H., Schimpl A., Feller A. C., Horak I. (1993). Ulcerative colitis-like disease in mice with a disrupted interleukin-2 gene.. Cell.

[PDF_00831] Sasaki T., Hiwatashi N., Yamazaki H., Noguchi M., Toyota T. (1992). The role of interferon gamma in the pathogenesis of Crohn's disease.. Gastroenterol Jpn.

[PDF_00637] Schreiber S., MacDermott R. P., Raedler A., Pinnau R., Bertovich M. J., Nash G. S. (1991). Increased activation of isolated intestinal lamina propria mononuclear cells in inflammatory bowel disease.. Gastroenterology.

[PDF_00688] Schröder J. M., Mrowietz U., Morita E., Christophers E. (1987). Purification and partial biochemical characterization of a human monocyte-derived, neutrophil-activating peptide that lacks interleukin 1 activity.. J Immunol.

[PDF_00844] Snapper C. M., Finkelman F. D., Paul W. E. (1988). Differential regulation of IgG1 and IgE synthesis by interleukin 4.. J Exp Med.

[PDF_00745] Stevens C., Walz G., Singaram C., Lipman M. L., Zanker B., Muggia A., Antonioli D., Peppercorn M. A., Strom T. B. (1992). Tumor necrosis factor-alpha, interleukin-1 beta, and interleukin-6 expression in inflammatory bowel disease.. Dig Dis Sci.

[PDF_00628] Strober W. (1985). Animal models of inflammatory bowel disease--an overview.. Dig Dis Sci.

[PDF_00723] Ueno Y., Takano N., Kanegane H., Yokoi T., Yachie A., Miyawaki T., Taniguchi N. (1989). The acute phase nature of interleukin 6: studies in Kawasaki disease and other febrile illnesses.. Clin Exp Immunol.

[PDF_00789] Urban J. F., Madden K. B., Svetić A., Cheever A., Trotta P. P., Gause W. C., Katona I. M., Finkelman F. D. (1992). The importance of Th2 cytokines in protective immunity to nematodes.. Immunol Rev.

[PDF_00686] Vilcek J., Lee T. H. (1991). Tumor necrosis factor. New insights into the molecular mechanisms of its multiple actions.. J Biol Chem.

[PDF_00731] Waage A., Brandtzaeg P., Halstensen A., Kierulf P., Espevik T. (1989). The complex pattern of cytokines in serum from patients with meningococcal septic shock. Association between interleukin 6, interleukin 1, and fatal outcome.. J Exp Med.

[PDF_00743] Woloski B. M., Smith E. M., Meyer W. J., Fuller G. M., Blalock J. E. (1985). Corticotropin-releasing activity of monokines.. Science.

[PDF_00784] Yamamura M., Uyemura K., Deans R. J., Weinberg K., Rea T. H., Bloom B. R., Modlin R. L. (1991). Defining protective responses to pathogens: cytokine profiles in leprosy lesions.. Science.

[PDF_00840] Yokota T., Otsuka T., Mosmann T., Banchereau J., DeFrance T., Blanchard D., De Vries J. E., Lee F., Arai K. (1986). Isolation and characterization of a human interleukin cDNA clone, homologous to mouse B-cell stimulatory factor 1, that expresses B-cell- and T-cell-stimulating activities.. Proc Natl Acad Sci U S A.

[PDF_00698] Youngman K. R., Simon P. L., West G. A., Cominelli F., Rachmilewitz D., Klein J. S., Fiocchi C. (1993). Localization of intestinal interleukin 1 activity and protein and gene expression to lamina propria cells.. Gastroenterology.

